# *N*-(2-Pyridylmethanimidamido)pyridine-2-carboximidamide

**DOI:** 10.1107/S1600536810016351

**Published:** 2010-05-29

**Authors:** Yingchun Wang

**Affiliations:** a Ordered Matter Science Research Center, Southeast University, Nanjing 210096, People’s Republic of China

## Abstract

In the title mol­ecule, C_12_H_12_N_6_, the dihedral angles between the pyridine rings and the central dimethan­imine–hydrazine group are 0.30 (3) and 13.94 (3)°. Two intra­molecular N—H⋯N hydrogen bonds stabilize the planar conformation of one pyridine ring with respect to its hydrazine-residue neighbour, whereas the other pyridine ring and an N-bonded H atom are rotated out of the plane and link the mol­ecules into inter­molecular N—H⋯N chains propagating in [010].

## Related literature

For the phase transition of pyridinium tetra­chloro­iodate(III) studied by X-ray analysis and dielectric and heat capacity measurements, see: Asaji *et al.* (2007 ▶). For the synthesis of 2-pyridylpyridines *via* Diels–Alder reactions between 3-pyridyl-1,2,4-triazines and vinyl­alcanoates, see: Shintou *et al.* (2005 ▶). For the ferroelecric properties of pyridinum perrhenate, see: Wasicki *et al.* (1997 ▶).
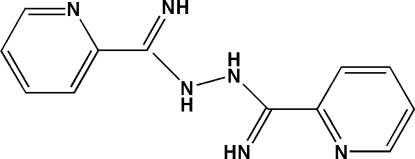


## Experimental

### 

#### Crystal data

C_12_H_12_N_6_
*M* *_r_* = 240.28
Orthorhombic, 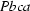

*a* = 13.218 (3) Å
*b* = 9.4979 (19) Å
*c* = 19.811 (4) Å
*V* = 2487.2 (9) Å^3^
*Z* = 8
Mo *K*α radiation
μ = 0.09 mm^−1^
*T* = 293 K
0.20 × 0.20 × 0.20 mm

#### Data collection

Rigaku SCXmini diffractometer
Absorption correction: multi-scan (*CrystalClear*; Rigaku, 2005 ▶) *T*_min_ = 0.5, *T*_max_ = 0.5
23997 measured reflections
2848 independent reflections
1934 reflections with *I* > 2σ(*I*)
*R*_int_ = 0.075

#### Refinement

*R*[*F* ^2^ > 2σ(*F* ^2^)] = 0.073
*wR*(*F* ^2^) = 0.222
*S* = 1.09
2848 reflections
168 parameters
2 restraints
H atoms treated by a mixture of independent and constrained refinement
Δρ_max_ = 0.46 e Å^−3^
Δρ_min_ = −0.67 e Å^−3^

### 

Data collection: *CrystalClear* (Rigaku, 2005 ▶); cell refinement: *CrystalClear*; data reduction: *CrystalClear*; program(s) used to solve structure: *SHELXS97* (Sheldrick, 2008 ▶); program(s) used to refine structure: *SHELXL97* (Sheldrick, 2008 ▶); molecular graphics: *SHELXTL* (Sheldrick, 2008 ▶); software used to prepare material for publication: *PRPKAPPA* (Ferguson, 1999 ▶).

## Supplementary Material

Crystal structure: contains datablocks I, global. DOI: 10.1107/S1600536810016351/si2254sup1.cif

Structure factors: contains datablocks I. DOI: 10.1107/S1600536810016351/si2254Isup2.hkl

Additional supplementary materials:  crystallographic information; 3D view; checkCIF report

## Figures and Tables

**Table 1:** Hydrogen-bond geometry (Å, °)

*D*—H⋯*A*	*D*—H	H⋯*A*	*D*⋯*A*	*D*—H⋯*A*
N4—H4*B*⋯N5^i^	0.86	2.60	3.096 (3)	117
N2—H2*B*⋯N1	0.79 (4)	2.34 (4)	2.670 (4)	106 (3)
N3—H3*B*⋯N5	0.86	2.33	2.619 (3)	100
N4—H4*B*⋯N2	0.86	2.31	2.608 (3)	101

Symmetry code: (i) 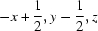.

**Fig. 1.**
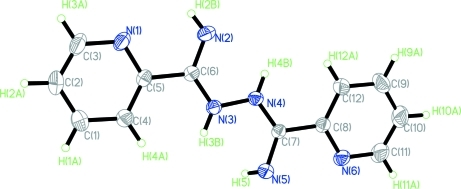
The molecular structure of the title compound, with the atomic numbering scheme. Displacement ellipsoids are drawn at the 30% probability level.

**Fig. 2.**
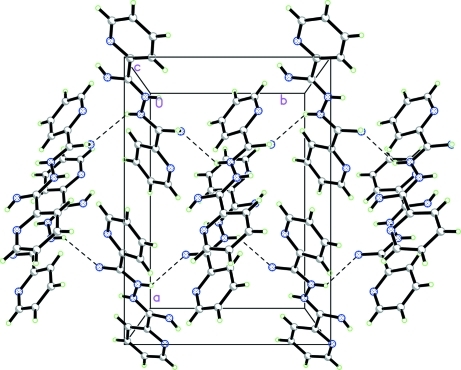
A view of the packing of the title compound, stacking along the c axis. Dashed lines indicate hydrogen bonds.

## supplementary crystallographic information

### Comment

The study of seignette-electrics materials has received much attention. Some materials have predominant dielectric-ferroelectric performance. The study of phase transition and related dielectric-ferroelectric property about PyHX (X=ICl_4_, ClO_4_, IO_4_, ReO_4_etc) (Asaji *et al.* (2007); Wasicki *et al.* (1997)) has received much attention. As one part of our continuing studies on finding for dielectric-ferroelectric materials, especially which contain N—H···N hydrogen bonds, we synthesized the title compound C_12_H_12_N_6_(I). It has no phase-transition in dielectric measurement during 93 K to 425 K (m.p 458 K).

The compound contains approximate non-crystallographic inversion symmetry (Fig 1). The torsion angles of N2—C6—C5—N1 and N4—N3—C6—C5 are 3.3 (3)° and 179.12 (19)°, N3—C6—C5—N1 and N4—N3—C6—N2 are -176.7 (2)° and -0.9 (4)°, C7—N4—N3—C6 is 174.0 (2)°. H5 rotates out of the molecular plane to prevent collision with the H4B of the intermolecular hydrogen N4—H4B···N5^i^ bond. Two intramolecular hydrogen bonds (N2—H2B···N1 and N4—H4B···N2) contribute to the planar conformation of the N1 pyridine with the dimethanimine-hydrazine group. The other pyridine unit rotates out of the central hydrazine by 13.94 (3)° because N5—H5···N6 intramolecular bond is not realized. The intermolecular hydrogen bonds (N4—H4B···N5^i^, Table 1) link the molecules into chains along the b-axis (Fig 2).

### Experimental

Picolinonitrile 5.2 g (100 mmol) and hydrazine hydrate 2.94 g (85%, 100 mmol) in flask and water (75 ml) was added, then the reagent react at 50°C for 24 h (Shintou  *et al.*(2005)). The reaction solution was extracted by dichloromethane, and the solvate was removed under reduced pressure and the product was obtained as yellow solid. The crystals suitable for structure determination were grown by slow evaporation in dichloromethane and methanol (1: 1) at room temperature.

### Refinement

Positional parameters of all the H atoms were calculated geometrically and were allowed to ride on the C atoms to which they are bonded,with C—H = 0.93Å , N—H = 0.75-0.86 Å; with *U*_iso_(H) = 1.2U_eq_(C), and with *U*_iso_(H) = 1.2-1.5U_eq_(N).

### Crystal data

**Table:**

C_12_H_12_N_6_	*F*(000) = 1008
*M**_r_* = 240.28	*D*_x_ = 1.283 Mg m^−^^3^
Orthorhombic, *P**b**c**a*	Mo *K*α radiation, λ = 0.71073 Å
Hall symbol: -P 2ac 2ab	Cell parameters from 8973 reflections
*a* = 13.218 (3) Å	θ = 3.0–8973°
*b* = 9.4979 (19) Å	µ = 0.09 mm^−^^1^
*c* = 19.811 (4) Å	*T* = 293 K
*V* = 2487.2 (9) Å^3^	Prism, colorless
*Z* = 8	0.20 × 0.20 × 0.20 mm

### Data collection

**Table:**

Rigaku SCXmini diffractometer	2848 independent reflections
Radiation source: fine-focus sealed tube	1934 reflections with *I* > 2σ(*I*)
graphite	*R*_int_ = 0.075
Detector resolution: 13.6612 pixels mm^-1^	θ_max_ = 27.5°, θ_min_ = 3.1°
ω scans	*h* = −17→17
Absorption correction: multi-scan (*CrystalClear*; Rigaku, 2005)	*k* = −12→12
*T*_min_ = 0.5, *T*_max_ = 0.5	*l* = −25→25
23997 measured reflections	

### Refinement

**Table:**

Refinement on *F*^2^	Primary atom site location: structure-invariant direct methods
Least-squares matrix: full	Secondary atom site location: difference Fourier map
*R*[*F*^2^ > 2σ(*F*^2^)] = 0.073	Hydrogen site location: inferred from neighbouring sites
*wR*(*F*^2^) = 0.222	H atoms treated by a mixture of independent and constrained refinement
*S* = 1.09	*w* = 1/[σ^2^(*F*_o_^2^) + (0.1052*P*)^2^ + 1.2299*P*] where *P* = (*F*_o_^2^ + 2*F*_c_^2^)/3
2848 reflections	(Δ/σ)_max_ < 0.001
168 parameters	Δρ_max_ = 0.46 e Å^−^^3^
2 restraints	Δρ_min_ = −0.67 e Å^−^^3^

### Special details

**Table:**

Geometry. All esds (except the esd in the dihedral angle between two l.s. planes) are estimated using the full covariance matrix. The cell esds are taken into account individually in the estimation of esds in distances, angles and torsion angles; correlations between esds in cell parameters are only used when they are defined by crystal symmetry. An approximate (isotropic) treatment of cell esds is used for estimating esds involving l.s. planes.
Refinement. Refinement of *F*^2^ against ALL reflections. The weighted *R*-factor wR and goodness of fit *S* are based on *F*^2^, conventional *R*-factors *R* are based on *F*, with *F* set to zero for negative *F*^2^. The threshold expression of *F*^2^ > σ(*F*^2^) is used only for calculating *R*-factors(gt) etc. and is not relevant to the choice of reflections for refinement. *R*-factors based on *F*^2^ are statistically about twice as large as those based on *F*, and *R*- factors based on ALL data will be even larger.

### Fractional atomic coordinates and isotropic or equivalent isotropic displacement parameters (Å^2^)

**Table:**

	*x*	*y*	*z*	*U*_iso_*/*U*_eq_	
N4	0.15435 (14)	0.0172 (2)	0.56008 (10)	0.0402 (5)	
H4B	0.1516	−0.0618	0.5390	0.048*	
N3	0.09172 (14)	0.0508 (2)	0.61420 (10)	0.0411 (5)	
H3B	0.0979	0.1263	0.6379	0.049*	
C6	0.02347 (17)	−0.0432 (2)	0.62501 (12)	0.0397 (6)	
C7	0.21721 (16)	0.1167 (2)	0.54472 (12)	0.0361 (5)	
C8	0.28736 (18)	0.0908 (2)	0.48714 (12)	0.0408 (6)	
C5	−0.04819 (17)	−0.0210 (3)	0.68259 (13)	0.0421 (6)	
C4	−0.0456 (2)	0.0985 (3)	0.72202 (14)	0.0501 (7)	
H4A	0.0019	0.1687	0.7138	0.060*	
N5	0.22387 (18)	0.2410 (2)	0.57686 (13)	0.0556 (7)	
H5	0.2221	0.2290	0.6143	0.083*	
N6	0.36735 (17)	0.1762 (2)	0.48319 (13)	0.0570 (7)	
N1	−0.11358 (18)	−0.1259 (3)	0.69313 (13)	0.0622 (7)	
N2	0.0109 (2)	−0.1617 (3)	0.58838 (14)	0.0637 (8)	
C12	0.2684 (2)	−0.0139 (3)	0.44062 (14)	0.0549 (7)	
H12A	0.2111	−0.0701	0.4442	0.066*	
C1	−0.1144 (2)	0.1123 (4)	0.77361 (15)	0.0594 (8)	
H1A	−0.1142	0.1921	0.8008	0.071*	
C11	0.4310 (3)	0.1556 (4)	0.4320 (2)	0.0815 (11)	
H11A	0.4872	0.2141	0.4284	0.098*	
C9	0.3363 (3)	−0.0335 (4)	0.38862 (17)	0.0737 (10)	
H9A	0.3263	−0.1046	0.3570	0.088*	
C2	−0.1828 (2)	0.0077 (4)	0.78440 (17)	0.0668 (9)	
H2A	−0.2307	0.0154	0.8186	0.080*	
C10	0.4181 (3)	0.0526 (4)	0.38424 (19)	0.0836 (12)	
H10A	0.4647	0.0419	0.3494	0.100*	
C3	−0.1799 (2)	−0.1083 (4)	0.7442 (2)	0.0771 (10)	
H3A	−0.2264	−0.1797	0.7524	0.093*	
H2B	−0.035 (3)	−0.213 (4)	0.596 (2)	0.094 (14)*	

### Atomic displacement parameters (Å^2^)

**Table:**

	*U*^11^	*U*^22^	*U*^33^	*U*^12^	*U*^13^	*U*^23^
N4	0.0408 (11)	0.0333 (10)	0.0464 (11)	−0.0003 (8)	0.0028 (9)	−0.0029 (9)
N3	0.0382 (10)	0.0371 (11)	0.0480 (12)	−0.0019 (9)	0.0041 (9)	−0.0033 (9)
C6	0.0343 (11)	0.0377 (12)	0.0472 (13)	0.0026 (10)	−0.0009 (10)	0.0029 (10)
C7	0.0352 (11)	0.0328 (11)	0.0404 (12)	0.0027 (9)	0.0015 (9)	0.0039 (9)
C8	0.0428 (13)	0.0333 (12)	0.0464 (13)	0.0046 (10)	0.0036 (10)	0.0045 (10)
C5	0.0354 (12)	0.0433 (13)	0.0474 (13)	0.0030 (10)	0.0020 (10)	0.0079 (11)
C4	0.0458 (14)	0.0514 (15)	0.0532 (15)	0.0018 (12)	0.0002 (12)	0.0005 (13)
N5	0.0655 (15)	0.0419 (13)	0.0594 (14)	−0.0072 (11)	0.0184 (12)	−0.0095 (10)
N6	0.0511 (13)	0.0472 (13)	0.0727 (16)	−0.0064 (11)	0.0222 (11)	−0.0075 (11)
N1	0.0541 (14)	0.0555 (15)	0.0771 (17)	−0.0111 (11)	0.0220 (12)	0.0025 (12)
N2	0.0590 (15)	0.0525 (15)	0.0797 (18)	−0.0209 (13)	0.0172 (14)	−0.0130 (13)
C12	0.0655 (18)	0.0471 (15)	0.0520 (15)	−0.0007 (13)	0.0043 (13)	−0.0019 (13)
C1	0.0573 (17)	0.0675 (19)	0.0534 (16)	0.0132 (15)	0.0033 (13)	−0.0029 (14)
C11	0.071 (2)	0.071 (2)	0.102 (3)	−0.0065 (18)	0.047 (2)	−0.009 (2)
C9	0.102 (3)	0.0601 (19)	0.0585 (19)	0.015 (2)	0.0157 (18)	−0.0142 (15)
C2	0.0563 (17)	0.083 (2)	0.0610 (19)	0.0120 (16)	0.0198 (14)	0.0093 (17)
C10	0.091 (3)	0.076 (2)	0.085 (3)	0.009 (2)	0.049 (2)	−0.005 (2)
C3	0.0621 (19)	0.078 (2)	0.091 (3)	−0.0125 (17)	0.0332 (19)	0.006 (2)

### Geometric parameters (Å, °)

**Table:**

N4—C7	1.295 (3)	N6—C11	1.333 (4)
N4—N3	1.392 (3)	N1—C3	1.349 (4)
N4—H4B	0.8600	N2—H2B	0.79 (4)
N3—C6	1.287 (3)	C12—C9	1.379 (4)
N3—H3B	0.8600	C12—H12A	0.9300
C6—N2	1.350 (3)	C1—C2	1.360 (4)
C6—C5	1.498 (3)	C1—H1A	0.9300
C7—N5	1.344 (3)	C11—C10	1.371 (5)
C7—C8	1.490 (3)	C11—H11A	0.9300
C8—N6	1.335 (3)	C9—C10	1.359 (5)
C8—C12	1.379 (4)	C9—H9A	0.9300
C5—N1	1.336 (3)	C2—C3	1.360 (5)
C5—C4	1.378 (4)	C2—H2A	0.9300
C4—C1	1.374 (4)	C10—H10A	0.9300
C4—H4A	0.9300	C3—H3A	0.9300
N5—H5	0.7500		
			
C7—N4—N3	113.29 (19)	C5—N1—C3	116.4 (3)
C7—N4—H4B	123.4	C6—N2—H2B	121 (3)
N3—N4—H4B	123.4	C8—C12—C9	118.6 (3)
C6—N3—N4	112.7 (2)	C8—C12—H12A	120.7
C6—N3—H3B	123.7	C9—C12—H12A	120.7
N4—N3—H3B	123.7	C2—C1—C4	119.1 (3)
N3—C6—N2	125.2 (2)	C2—C1—H1A	120.4
N3—C6—C5	118.2 (2)	C4—C1—H1A	120.4
N2—C6—C5	116.7 (2)	N6—C11—C10	123.5 (3)
N4—C7—N5	124.9 (2)	N6—C11—H11A	118.3
N4—C7—C8	117.3 (2)	C10—C11—H11A	118.3
N5—C7—C8	117.8 (2)	C10—C9—C12	118.9 (3)
N6—C8—C12	122.9 (2)	C10—C9—H9A	120.5
N6—C8—C7	115.9 (2)	C12—C9—H9A	120.5
C12—C8—C7	121.2 (2)	C3—C2—C1	118.7 (3)
N1—C5—C4	122.8 (2)	C3—C2—H2A	120.6
N1—C5—C6	115.0 (2)	C1—C2—H2A	120.6
C4—C5—C6	122.2 (2)	C9—C10—C11	119.0 (3)
C1—C4—C5	119.0 (3)	C9—C10—H10A	120.5
C1—C4—H4A	120.5	C11—C10—H10A	120.5
C5—C4—H4A	120.5	N1—C3—C2	123.9 (3)
C7—N5—H5	109.5	N1—C3—H3A	118.0
C11—N6—C8	117.1 (3)	C2—C3—H3A	118.0
			
C7—N4—N3—C6	174.0 (2)	C12—C8—N6—C11	−0.9 (4)
N4—N3—C6—N2	−0.9 (4)	C7—C8—N6—C11	−179.7 (3)
N4—N3—C6—C5	179.12 (19)	C4—C5—N1—C3	1.4 (4)
N3—N4—C7—N5	0.3 (3)	C6—C5—N1—C3	−179.3 (3)
N3—N4—C7—C8	−179.77 (18)	N6—C8—C12—C9	1.6 (4)
N4—C7—C8—N6	−163.2 (2)	C7—C8—C12—C9	−179.6 (3)
N5—C7—C8—N6	16.7 (3)	C5—C4—C1—C2	0.1 (4)
N4—C7—C8—C12	17.9 (3)	C8—N6—C11—C10	0.0 (6)
N5—C7—C8—C12	−162.2 (2)	C8—C12—C9—C10	−1.4 (5)
N3—C6—C5—N1	−176.7 (2)	C4—C1—C2—C3	0.9 (5)
N2—C6—C5—N1	3.3 (3)	C12—C9—C10—C11	0.5 (6)
N3—C6—C5—C4	2.7 (3)	N6—C11—C10—C9	0.2 (6)
N2—C6—C5—C4	−177.3 (2)	C5—N1—C3—C2	−0.3 (5)
N1—C5—C4—C1	−1.4 (4)	C1—C2—C3—N1	−0.8 (6)
C6—C5—C4—C1	179.4 (2)		

### Hydrogen-bond geometry (Å, °)

**Table:**

*D*—H···*A*	*D*—H	H···*A*	*D*···*A*	*D*—H···*A*
N4—H4B···N5^i^	0.86	2.60	3.096 (3)	117
N2—H2B···N1	0.79 (4)	2.34 (4)	2.670 (4)	106 (3)
N3—H3B···N5	0.86	2.33	2.619 (3)	100
N4—H4B···N2	0.86	2.31	2.608 (3)	101

Symmetry codes: (i) −*x*+1/2, *y*−1/2, *z*.

## References

[bb1] Asaji, T., Eda, K., Fujimori, H., Adachi, T., Shibusawa, T. & Oguni, M. (2007). *J. Mol. Struct.* **826**, 24–28.

[bb2] Ferguson, G. (1999). *PRPKAPPA* University of Guelph, Canada.

[bb3] Rigaku (2005). *CrystalClear* Rigaku Corporation, Tokyo, Japan.

[bb4] Sheldrick, G. M. (2008). *Acta Cryst.* A**64**, 112–122. 10.1107/S0108767307043930 18156677

[bb5] Shintou, T., Ikeuchi, F., Kuwabara, H., Umihara, K. & Itoh, I. J. (2005). *Chemistry Lett.* **34**, 836–838.

[bb6] Wasicki, J., Czarnecki, P., Pajak, Z., Nawrocik, W. & Szepanski, W. (1997). *J. Chem. Phys* **107**, 576–578.

